# Nomenclatural Synopsis of *Cirsium* Sect. *Eriolepis* (Asteraceae) in Italy

**DOI:** 10.3390/plants10020223

**Published:** 2021-01-24

**Authors:** Emanuele Del Guacchio, Liliana Bernardo, Paolo Caputo, Francesca Carucci, Gianniantonio Domina, Duilio Iamonico

**Affiliations:** 1 Botanical Garden, University of Naples “Federico II”, 80139 Naples, Italy; edelgua@email.it (E.D.G.); paolo.caputo@unina.it (P.C.); francesca.carucci@hotmail.it (F.C.); 2 Department of Biology, Ecology and Earth Sciences, University of Calabria, 87036 Arcavacata di Rende, CS, Italy; liliana.bernardo@unical.it; 3 Department of Biology, University of Naples “Federico II”, 80139 Naples, Italy; 4 Department of Agricultural, Food and Forest Sciences, University of Palermo, 90128 Palermo, Italy; gianniantonio.domina@gmail.com; 5 PDTA Department, University of Rome Sapienza, 00196 Rome, Italy

**Keywords:** Carduinae, *Carduus*, *Cnicus*, Italian endemic plants, *Epitrachys*, Lacaita, L’Obel, Mediterranean flora, Petrak, Tenore, taxonomy, typification

## Abstract

The names of the Italian taxa in *Cirsium* sect. *Eriolepis* are discussed. The accepted names are: *Cirsium echinatum*, *C. eriophorum* subsp. eriophorum, *C. eriophorum* subsp. spathulatum, *C. ferox*, *C. italicum*, *C. lacaitae*, *C. lobelii*, *C. morisianum*, *C. scabrum*, *C. tenoreanum*, *C. vallis-demonii* subsp. vallis-demonii, *C. vallis-demonii* subsp. calabrum comb. nov., and *C. vulgare* (= *C. crinitum*, *C. sylvaticum*). Four accepted names are typified by specimens preserved at FI (one lectotype), G (one lectotype and one neotype), P (one lectotype), and by illustrations (two lectotypes). Several other heterotypic synonyms of taxa described from Italy are discussed and six of them are typified. A new combination and status are proposed: *C. vallis-demonii* subsp. calabrum, based on *C. eriophorum* var. vallis-demonii f. calabrum.

## 1. Introduction

*Cirsium* Mill. (Asteraceae Bercht. & J. Presl.: Cardueae Cass.) is a large genus comprising more than 450 species (as many as 491 according to POWO [1]), usually biennial or perennial spiny herbs, distributed in the northern hemisphere but also naturalized worldwide ([2,3,4]). Among the three sections currently recognized in Europe, sect. *Eriolepis* (Cass. in Cuvier) Dumort. (=*Ci.* sect. *Epitrachys* DC. ex Duby) is an extremely difficult group from the taxonomical point of view, and some taxa are only provisionally accepted ([5,6,7]. The members of this section are mostly biennial without vegetative multiplication, and they are characterized by pinnatifid and usually coriaceous leaves with segments divided into two divaricate basal lobes and with rigid setae (more properly, spines according to Keil [3]) on the upper surface of the blade, medium to large heads, middle involucral bracts with toothed margins, a narrow appendage abruptly contracted into one terminal and robust awn, corolla tube longer than limb (this latter more or less divided up to half), and pappus shorter to subequal than corolla (see e.g., [5,7,8]).

As a part of ongoing studies on the taxonomy of *Cirsium* sect. *Eriolepis* (see e.g., [9,10]) and on taxa endemic to central and southern Italy [11,12,13,14,15,16,17], we here present a nomenclatural contribution concerning the types of the names of the Italian taxa included in this section. To avoid confusions, the generic names *Carduus*, *Cirsium*, and *Cnicus* are abbreviated through the text as “*Ca.*”, “*Ci.*”, and “*Cn.*” respectively.

According to Greuter [6], the following taxa belonging to *Ci.* sect. *Eriolepis* occur in Italy: *Ci. echinatum* (Desf.) DC., *Ci. eriophorum* (L.) Scop., *Ci. ferox* (L.) DC., *Ci. italicum* DC., *Ci. lacaitae* Petr., *Ci. lobelii* Ten., *Ci. morisianum* Reich., *Ci. scabrum* (Poir.) Bonnet & Baratte, *Ci. spathulatum* (Moretti) Gaud. (considered by [18,19] as a subspecies of *Ci. eriophorum*), *Ci. tenoreanum* Petr., *Ci. vallis-demonii* Lojac., *Ci. vulgare* (Savi) Ten. subsp. *vulgare*, *Ci. vulgare* subsp. *crinitum* (Boiss. ex DC.) Arènes, and *Ci. vulgare* subsp. *silvaticum* (Tausch) Arènes. Four of these names (*Ci. echinatum*, *Ci. eriophorum*, *Ci. scabrum*, and *Ci. vulgare*) are combinations with basionyms in *Carduus* L.; two of them (*Ci. ferox* and *Ci. spathulatum*) are based on names in *Cnicus* L.

## 2. Material and Methods

This paper investigates the names in *Cirsium* sect. *Eriolepis* occurring in Italy and those published on Italian material, based on both analysis of the relevant literature (protologues included) and checking and/or examination of specimens kept at BOLO, BM, CAT, FI, G, H, K, LY, MS, NAP, OHN, P, PAD, PAL, PAV, PI, PRC, and RO [20]. The articles cited through the text belong to the *Shenzen Code* [21].

Within the group of our interest, numerous “microspecies” were published by Gandoger [22] under *Eriolepis*, together with new intended combinations, e.g., “*Eriolepis apennina*”, “*E. aprutia*”, “*E. apula*”, “*E. atrorubens*”, “*E. brevispina*”, “*E. brutia*”, “*E. calabrica*”, “*E. incerta*”, “*E. insubrica*”, “*E. lacerans*”, “*E. lancifera*”, “*E. leiantha*”, “*E. leptacantha*”, “*E. lobelii*”, “*E. majellensis*”, “*E. megachlamys*”, “*E. messanensis*”, “*E. misilmerensis*”, “*E. nebrodensis*”, “*E. nigricans*”, “*E. parva*”, “*E. recedens*”, “*E. secundaria*”, “*E. sicula*”, “*E. subpatens*”, “*E. tenuis*”, and “*E. tyrolensis*”. These names were generally not used later by Gandoger himself [23]. [24] attempted a synonimization of some of them. However, none of those names was validly published and they do not require any nomenclatural act [21]. Therefore, we excluded them from the present account.

## 3. Typification of the Names and Taxonomic Treatment

The taxonomic treatment generally follows [5], with the exception of *Ci. vallis-demonii* (which we split into two subspecies). Taxonomical notes are provided within each entry to justify our choice. In the following account, the accepted names are in alphabetical order; within each of them, the treated homotypic synonyms are listed in chronological order.

**Figure 1:**
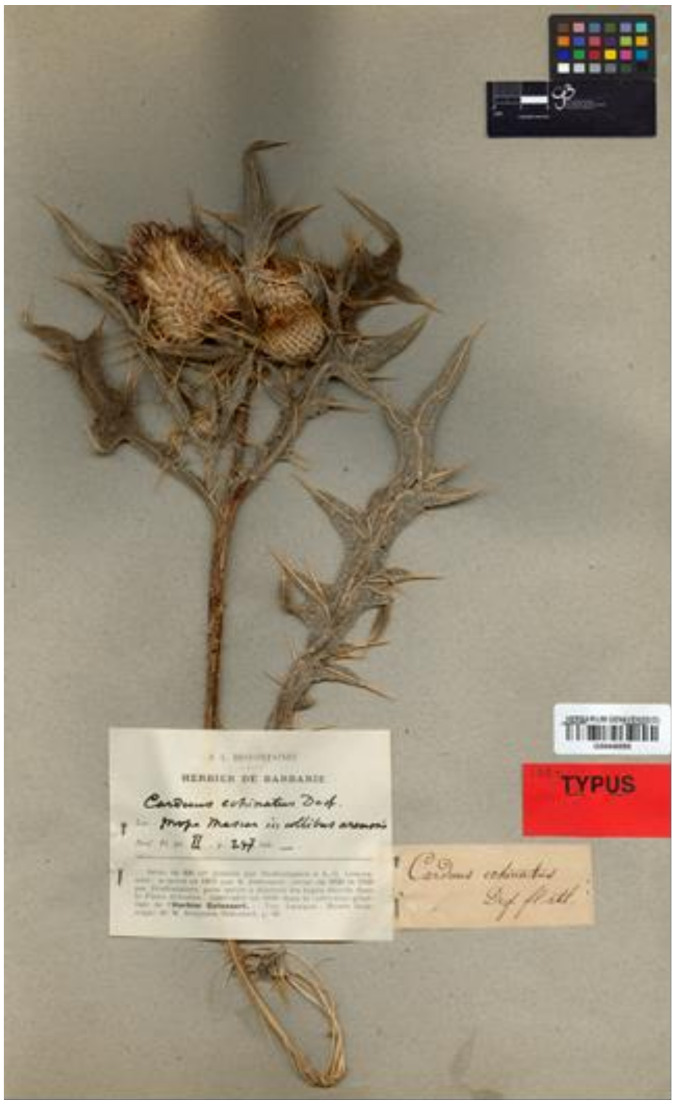
Lectotype of Ca. *echinatus* Desf. (G), by permission of the Curator.

**(1) *Cirsium echinatum*** (Desf.) DC., Fl. Franc., ed. 3, 6: 465. 1815 ≡ *Carduus echinatus* Desf., Fl. Atlant. 2: 247. 1799 (basion.) ≡ *Cnicus echinatus* Willd., Sp. Pl., ed. 4, 3(3): 1668. 1803.—Lectotype (designated by Talavera & Valdès [25] (p. 214)): Algeria. “Prope Mascar, in collibus arenosis”, s.d., *[R.] Desfontaines s.n.* (G00446590 [digital image!]).—Figure 1.

*Distribution*—Species endemic to western Mediterranean (Tunisia, Algeria, Morocco, Spain, France and Sicily) [6].

*Habitat*—Dry, open habitats in the Mediterranean area, on sandy or claysh soils, often basic and nitrified, up to 2100 m a.s.l. [8,18,26].

*Note*—The combinations “*Eriolepis echinata*”, “*Eriolepis italica*”, and “*Eriolepis ferox*” are sometimes attributed to Cassini [27], who actually wrote in [28] (p. 470): “Le *Cnicus ferox* de Linné, […] le *Cirsium echinatum* de M. De Candolle (Flor. franc., Suppl.), et le *Cirsium italicum* du même botaniste (*Cat. Hort. monsp.*), appartiennent à notre genre ou sous-genre *Eriolepis*”. Nevertheless, since Cassini (in [28]) did not definitively associate the epithets *ferox*, *echinatum* or *italicum* to *Eriolepis*, these combinations were not validly published (Art. 35.2).

**(2) *Cirsium eriophorum*** (L.) Scop., Fl. Carniol., ed. 2, 2: 130. 1771 ≡ *Carduus eriophorus* L. (basion.), Sp. Pl. 2: 893. 1753 ≡ *Cnicus eriophorus* (L.) Roth, Tent. Fl. Germ.: 345. 1788 ≡ *Eriolepis lanigera* Cass. in Cuvier, Dict. Sci. Nat. 41: 331. 1826, nom. illeg. (Art. 11.4).—Lectotype (designated by Del Guacchio & Iamonico [9] (p. 197)): Herb. Linnaeus, no. 966.32 (LINN [digital image!]).—http://linnean-online.org/9831/.“*Ci. eriophorum* var. *vulgare* Naeg.”, Syn. Fl. Germ. Helv., ed. 2, 3: 989. 1845, nom. inval. (Art. 26.2).“*Ci. eriophorum* subsp. *eu-eriophorum* var. *genuinum* Gillot”, Rev. Bot. 12: 360. 1894, nom. inval. (Art. 24.3).“*Ci. eriophorum* subsp. *vulgare* Petr.”, Biblioth. Bot. 78: 15. 1912, nom. inval. (Art. 26.2).

*Notes on* Ci. eriophorum *var.* vulgare—The final epithet “*vulgare*” was associated by Naegelius [29] (p. 989) to “*C. eriophorum* Auct.”. Among these authors, Naegelius [29] cited the Linnaean basionym under *Carduus*, and other combinations based on it. For this reason, the name clearly purports to indicate the taxon containing the type of the name of the next higher-ranked taxon, i.e., *Ci. eriophorum*. Therefore, in our opinion, Art. 26.2 must be applied and the name is to be regarded as invalidly published. Analogous reasoning can be applied for the name by Petrak ([24], p. 15), which cannot be formally based on that by Naegelius [29].

*Distribution*—Europe (from Spain to Romania, including Great Britain), and western Asia (Turkey); naturalized in Ireland [6,30,31].

*Habitat*—Mountain pastures, grasslands, wood margins and sometimes disturbed environments of temperate European climate, up to 2500 m a.s.l. (rarely below 600 m), on rich soils over limestone and chalk [8,30].

**(3)*Cirsium ferox*** (L.) DC. in Lamarck & Candolle, Fl. Franç., ed. 3, 4: 120. 1805 ≡ *Cnicus ferox* L., Mant. Pl.: 109. 1767 ≡ *Carthamus ferox* (L.) Lam., Fl. Franç. (Lamarck) 2: 11. 1779 ≡ *Ca. ferox* (L.) Vill., Prosp. Hist. Pl. Dauphiné: 30. 1779 ≡ *Eriolepis ferox* (L.) Fourr., Ann. Soc. Linn. Lyon, sér. 2, 17: 111. 1869 ≡ *Cirsium eriophorum* var. *ferox* (L.) Fiori, Fl. Italia [Fiori, Béguinot and Paoletti] 3: 367. 1904 (sub var. “*ferox* (DC.)”).—Lectotype (designated by Del Guacchio and Iamonico ([9], p. 198)): [illustration] Carduus lanceolatus ferocior in [32] (p. 58).—http://bibdigital.rjb.csic.es/ing/Libro.php?Libro=4182&Hojas.

*Distribution*—Species endemic to eastern Spain, southern France and North-western Italy [6,8,19]. *Habitat*:—Dry open habitats, pastures, roadsides, stony slopes in the Mediterranean area, up to 1500 m a.s.l. [8].

*Note on* Carthamus ferox—Lamarck [33] does not cite the basionym *Cn. ferox* L., but we think that in this case Art. 41.4 can be applied, cf. [34] (p. 120).

*Note on* Eriolepis ferox—For the presumed identical combination by Cassini (e.g., [27]), see the note to *Ci. echinatum*.

**(4) *Cirsium italicum*** DC., Cat. Pl. Horti Monsp.: 96. 1813 ≡ *Carduus italicus* (DC.) Savi, Bot. Etrusc. 3: 140. 1818 ≡ *Cnicus italicus* (DC.) Sebast. & Mauri, Fl. Roman. Prodr.: 282. 1818.—Lectotype (designated here): Italy, “Entre Vallombrosa et Camaldoli”, 18 August 1808, *A. P. Candolle s.n.* [(G-DC00486549 [digital image!]).—http://www.ville-ge.ch/musinfo/bd/cjb/chg/adetail.php?id=335990&base=img&lang=fr).

**Figure 2:**
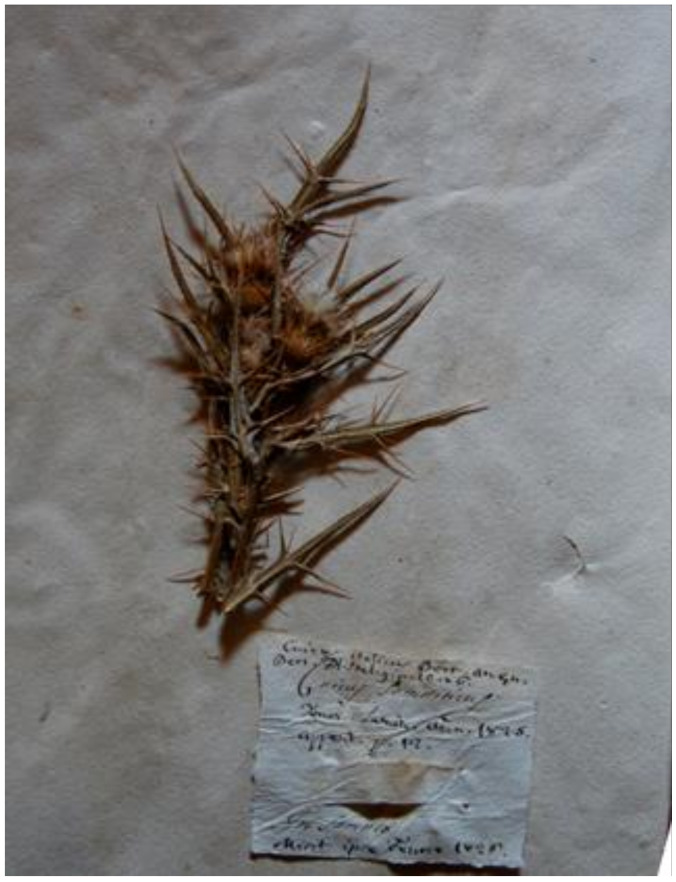
Neotype of *Cn. samniticus* Ten. (BOLO), by permission of the Curator.

=*Cnicus samniticus* Ten.—Neotype (designated here): “In Samnio”, s.d. [1825?], s.c., *s.n.* (BOLO [digital image!]).—Figure 2.

*Notes on* Ci. italicum—Candolle [35] published *Ci. italicum* with a detailed Latin description, with the provenance (“In ruderatis Etruriae et agri Romani”; transl.: “In ruderal environments of Tuscany and the surroundings of Rome”), and a taxonomic note. Note that according to CHG [36] several specimens in G-DC from Tuscany (as that of *Ci. italicum*) were indicated as collected by Candolle himself, but we have not further proof of this statement. Candolle [35] cited also a synonym from L’Obel [37] (p. 15) (“Phoenix leo carduus ferox”) and the illustrations of this plant by the same author (“p. 15 Figure 2”), by Dodoens [38] (p. 738), by Dalechamps [39] (p. 1489), and by Bauhin & Cherler [32] (p. 92). All these images are original material for the name *Ci. italicum*; however, Dodoens [38] and Dalechamps [39] reproduced the same figure by L’Obel [40], while that by Bauhin & Cherler [32] appears as a simplification of it [in fact, these authors report the description by L’Obel [40]. For simplicity’s sake, we refer by “L’Obel (1581)” to the first edition of the illustrations excerpted from L’Obel [40], that is “traditionally, but erroneously attributed to Lobel” [41]. Similarly, we indicate by “L’Obel (1591)” the second edition of the same work. We also found one specimen at G-DC (G00486549, image available at http://www.ville-ge.ch/musinfo/bd/cjb/chg/adetail.php?id=335990&base=img&lang=fr) that bears two flowered and fruiting stems (of possibly different individuals), collected by Candolle in Tuscany in 1808. The features of the exsiccatum match Candolle’s diagnosis: leaves shortly decurrent (i.e., stems partly winged), pinnatifid, tomentose below and roughly setose above, heads almost sessile and surrounded by the upper leaves, involucral bracts glabrescent, appressed and ending into a spine. According to Davis & Parris [42], this is the only member of the section showing vittae (in this case, a median rib on the bract). Besides, the label data fit the protologue information well. We designate this G-DC specimen as the lectotype of the name *Ci. italicum*. Our choice fully supports the current usage of the name, well deserved for a species endemic to North-Eastern Mediterranean: Corse, Italy (Sardinia and Sicily included), Malta, Albania, Greece and Turkey [6]. For a presumed combination “*Ci. italicum* (Savi) DC.” [5], see [43]; for “*Eriolepis italica* Cass.”, see our note to *Ci. echinatum*.

*Notes on* Cn. samniticus—Tenore [44] described *Ci. samniticus* in Latin and indicated the following provenance: “Habitat in montibus Samnii”, i.e., “on the mounts of Sannio”, an area roughly corresponding to southern Abruzzo, the modern Molise and North-eastern Campania regions in southern Italy. The protologue is included in the catalogue of the seeds collected in 1825 [44] and it could have been therefore published in 1825 or even in 1826. The only useful exsiccatum preserved in the Tenore Collection (NAP) bears a label by the author without the date of collection (“*Cnicus samniticus*\*C. spinosissim*. Florae nap. prodr.\in Samnio”) and it is represented by an individual with flowers and ripe heads. The only other specimen that we were able to locate is preserved at BOLO: nowadays it is reduced to a single fragment of flowering scape, and collected “in Samnio”. This specimen was labelled by Tenore himself as *Cn. samniticus*. In 1826 Tenore sent this specimen to Bertoloni, who added on the same label the references and the accepted name, i.e., *Cn. italicus* (cf. [45], p. 10). In our opinion it could be considered as original material for the name, but a definitive proof is lacking. In any case, it fully matches the original description [44]. We here prudentially propose it as neotype according to the Art. 9.7. Nowadays, *Ci. samniticus* is regarded a heterotypic synonym of *Ci. italicum* [18], and we agree by examining the relevant specimens (stems partly winged; small and glabrescent heads crowded upwards and exceeded by the apical leaves; involucral bracts vittate with patent spines).

*Distribution*—Endemic to North-Eastern Mediterranean Basin, from France (Corse) to Asiatic Turkey; naturalized in Germany [6].

*Habitat*—Arid meadows, shrublands, uncultivated fields, roadsides, up to 1100 m a.s.l. [18].

**(5) *Cirsium lacaitae*** Petr., Österr. Bot. Z. 64: 456. 1914 ≡ *Cirsium stabianum* Lacaita, Nuovo Giorn. Bot. Ital. n.s. 25: 120. 1918, nom. illeg. (Art. 52).—Lectotype (designated by Del Guacchio et al. [10]): Italy “Scala (Salerno) in Monte Canalitto, solo pomiceo, c. 1260 m”, 22 September 1912, *C. Lacaita 14574*, rev.. Petrak 1913 (BM001043042 [digital image!]). https://data.nhm.ac.uk/dataset/collection-specimens/resource/05ff2255-c38a-40c9-b657-4ccb55ab2feb/record/1971435.

*Note on* Ci. stabianum—This superfluous name is homotypic with *Ci. lacaitae* (Art. 7.5).

*Distribution*—This is the rarest representative of the section in Italy, restricted to Campania (southern Italy); known for the Peninsula of Sorrento [46], where it had been re-discovered, it also occurs on Picentini Massif [47]. In addition, some reports of *Ci. morisianum* from the same region are probably to be referred to *Ci. lacaitae* as well (Del Guacchio, pers. obs.). In fact, in agreement with Lacaita [46], we think that *Ci. lacaitae* is mostly related to *Ci. morisianum*, and it could be a southern vicariant of it; however, no intermediate populations have been yet described.

*Habitat*—Beech and chestnut woods, clearings, usually on rich and mature soils, at about 600–1300 m a.s.l. [47].

**(6) *Cirsium lobelii*** Ten., Index Sem. Hort. Bot. Neapol. 1830: 16. 1830 ≡ *Ci. eriophorum* subsp. *lobelii* Rouy, Bull. Soc. Bot. France 51: 428. 1904 ≡ *Ci. eriophorum* var. *lobelii* (Ten.) Fiori, Fl. Italia [Fiori, Béguinot & Paoletti] 3: 367. 1904.—Lectotype (designated here): [illustration] “Phoenix. Leo. Carduus ferox” in L’Obel [40] (p. 15).—http://bibdigital.rjb.csic.es/ing/Libro.php?Libro=4362&Hojas.—Epitype (designated here): Italy, Abruzzo, Monte Morrone, August 1914, *D. Profeta s.n.* (P04277119 [Digital image!]).—http://mediaphoto.mnhn.fr/media/1441358086652IKEzLI1D5aQmeuQZ.

=*Cirsium morisianum* var. *aprutianum* Petr, Biblioth. Bot. 78: 15. 1912.—Lectotype (designated here): Italy, Abruzzi, “in pascuis saxosis montis Morrone et Majella in Valle Cupa et al.la Rapina”, “sol. cal.—m. 2000–400” [on calcareous soil at 2000–2400 m a.s.l.], August 1905, *G.* Rigo (P, P04316688 [digital image!], sub “Cirsium Moritzianum Reich.” [sic!]).—http://mediaphoto.mnhn.fr/media/14413587569952aN3OdAPGgtS3EPg.

-  *Cirsium ferox* L. var. *lobelii* sensu DC.

“*Ci. eriophorum* L. subsp. *odontolepis* (Boiss. ex DC.) Rouy var. *aprutianum* Rouy”, Bull. Soc. Bot. France 51: 428. 1904, nom. inval. (Art. 38.2 Ex.1).

*Notes on* Ci. lobelii—The protologue of this name [48], dedicated to the famed Flemish botanist Mathias de L’Obel (1538–1616), includes a Latin description and the same polynomial by L’Obel ([40], p. 15) himself, already cited by Candolle [35] for *Ci. italicum*. According to our research, the personal copy of Tenore was a second edition of the work (L. Paino, *pers. comm.*), and also the other two copies known in Naples (one lost) were 1591 editions. Indeed, Tenore [48] listed the same illustrations cited by Candolle, i.e., those by Dodoens ([38], p. 738), (“788” in the Tenore protologue), by Dalechamps ([39], p. 1489), and by Bauhin and Cherler ([32], p. 92). In fact, according to Tenore [48], all these illustrations (which are original material) could be referred to his *Ci. lobelii*, rather than to *Ci. italicum*. This statement is reliable, in our opinion (cf. also [46]). In fact, even if somewhat compatible with the illustration by L’Obel [37,40], *Ci. italicum* has semi-winged stems, what is not clearly shown in the figure. In the protologue [48], a diagnostic comparison with *Ci. ciliatum* (Murr.) Moench and *Ci. italicum* was also provided. Tenore [48] also proposed two unnamed varieties: “var. A” (“Caulis 1–2 pedalis apice tantum ramosus, flores 6–8 lin. diametri”; transl.: “Stem 30–60 cm tall, branched only at the apex, with heads 12–16 mm in diameter”), which should be intended as the typical one, and “var. B” (“Planta ramosissima omnibus partibus duplo major, spinis robustissimis horrida”; transl. “A very branched plant, twice larger in every part than the former variety, with very robust spines”). Lacking the varietal epithet, these two varieties were not validly published (Art. 32.1, Note 1). On the basis of the diagnoses, they appear as compatible with the taxa currently called *C. lobelii* and *C. lacaitae*, respectively, as indicated by Lacaita [46]. This latter author suggested that the original description of *Ci. lobelii* was drawn up by field samples, not preserved by Tenore afterwards. His very detailed account of all the specimens preserved at NAP and those sent by Tenore to other botanists (all gathered after the protologue’s publication), and of the numerous citations (or illustrations) occurring in the Tenore’s works, definitively clarifies that Tenore used the name *Ci. lobelii* for different taxa. Actually, we have not been able to trace any specimen identifiable as original material either in the Tenore’s Collection at NAP, or at FI, G, and RO. However, a specimen at BOLO, collected on the mounts of Sannio, was sent by Tenore himself to Bertoloni in 1830 under the name *Ci. lobelii*, and filed as *Cn. eriophorum* var. “*β*” by Bertoloni [43] (p. 26). A further specimen is at K (K000778040, http://apps.kew.org/herbcat/getImage.do?imageBarcode=K000778040). It was collected in Abruzzo, sent by Tenore to J. Gay on May 1830, and bears a label handwritten by Tenore himself. The sending dates strongly suggest that these specimens may be original material. The plants on both the sheets at BOLO and K are not the ones currently named *Ci. lobelii*, but actually *Ci. tenoreanum.* In fact, the heads are less wide than 30 mm, the involucral bracts are patent with a rhombic, typically purplish appendage, the tube is more or less equalling the limb. As a consequence, we designate the image by L’Obel [40] as the lectotype of the name *Ci. lobelii* to preserve the current use. Fortunately, this illustration matches the Tenore’s diagnosis, and also corresponds to the current concept of the related taxon (whose circumscription is largely based on the Lacaita’s concept). The taxon is endemic to Apennines (Italy) [5,18]. However, since the illustration is not univocally identifiable with C. lobelii [46], an epitype is to be chosen (Art. 9.8). We choose a specimen at P (04277119, http://mediaphoto.mnhn.fr/media/1441358086652IKEzLI1D5aQmeuQZ) collected in “Abruzzi” (*locus classicus*) and originated from the herbarium of Lacaita, who first limited the modern application of the name and reported the gathering of the epitype, with a photograph [46]) (p. 121, Plate II).

*Notes on* Ci. ferox *var.* lobelii—The Tenore name *Ci. lobelii* was used by Candolle [49] to propose the combination at the varietal rank under *Ci. ferox*. However, on the basis of Candolle’s description and the examination of a specimen sent to him by Tenore (“in Aprutio e Lucania…1833”, G-DC, code 00486593!), we deduce that, by his combination, Candolle actually indeed indicated *C. tenoreanum* (see also [46]). Nevertheless, the combination by Candolle has the same nomenclatural type of *Ci. lobelii*, and therefore it must be referred to the same taxon (Art. 48.1, Note 1). However, as first observed by Lacaita [46], it is rather surprising that Candolle [49], in the same work, identified two specimens of the same species with two different taxa, i.e., *Ci. ferox* var. *lobelii* and *Ci. eriophorum* var. *spurium*.

*Notes on* Ci. eriophorum *subsp.* lobelii—Rouy [50] (p. 428) proposed this combination applying Tenore’s basionym *Ci. lobelii* to *Ci. tenoreanum* (as most authors did) and erroneously indicating its presence also in Greece. Nevertheless, *Ci. eriophorum* subsp. *lobelii* is homotypic with *Ci. lobelii* (Art. 7.3).

*Notes on* Ci. eriophorum *subsp.* odontolepis *var.* aprutianum—By this name, Rouy [50] intended to indicate the plant nowadays named *Ci. lobelii*, as Lacaita [46] clarified on the basis of a specimen kept in the Gussone’s collection (NAP) and revised by Rouy. Unfortunately, this specimen cannot be anymore traced at NAP (R. Vallariello, *in litt.*). However, since the name was published without a diagnosis or description, or a reference to a former one (“*C. Lobelii* bot. ital. nonnull., *non* Ten.”), it is a nomen nudum and therefore invalid (Art. 38.2, Ex.1).

*Notes on* Ci. morisianum *var.* aprutianum—Petrak [24], when intended to transfer the invalid varietal name of Rouy [50] under *Ci. morisianum*, actually described a new variety (Art. 12). He provided a detailed description and a diagnosis (in Latin) to distinguish the variety from the typical *Ci. morisianum*, a rich list of syntypes, the native range (unfortunately confusing Calabria with Abruzzi), and an original illustration of the involucral bracts. Besides, he added some accurate notes in German language. Among the numerous syntypes, Petrak [24] cited no. 4142 of the series “Herbarium normale”, collected by Rigo in 1898 ([51], p. 42), under “Cirsium Boujarti”), and other material gathered by Rigo himself (in addition to the herbaria cited by Petrak, the syntypes are nowadays also preserved—for example—at FI, NAP, P and US). We propose a syntype gathered by Rigo in 1905, derived from the Herbarium of L. Giraudias as the lectotype. It was reported by Petrak [24] with minor inaccuracies and revised by him in 1910. The specimen is complete of basal leaves and of a stem with two heads, one flowered and the other one in fruit: it represents without doubt the typical *Ci. lobelii* as circumscribed by modern authors.

*Distribution*—Species endemic to central Italy, southward to Campania [19], where, however, its distribution would require verification (Del Guacchio, *pers. obs.*). For example, it does not occur in the Peninsula of Sorrento [46,52]), despite the statement by Pignatti [18].

*Habitat*—Mountain pastures, rocky slopes, screes, on limestones, from about 1000 m to 2000 m a.s.l. [18].

**(7) *Cirsium morisianum*** Rchb., Icon. Fl. Germ. Helv. 15: 59. 1853 ≡ *Cirsium eriophorum* var. *morisianum* (Rchb.f.) Fiori, Fl. Italia [Fiori, Béguinot and Paoletti] 3: 367. 1904 ≡ *Ci. eriophorum* subsp. *morisianum* (Rchb.f.) Briq. and Cavill. in Burnat Fl. Alp. Marit. 7: 19. 1931.—Lectotype (designated by Lacaita ([46], p. 131)): France, “In collibus aridissimis supra Tenda Carlinum versus inter *Genistam candicantem*”, 23 July 1843, *H. G. Reichenbach s.n.* (W).

*Notes on* Ci. morisianum—The protologue of *C. morisianum* [53] is composed of a Latin diagnosis, a description, and the details of the gathering on the hills above the town of Tenda, in South-eastern France at the border with Italy (“[…] in collibus aridissimis supra Tenda Carlinum versus […] 23. Jul. 1843 […] Rchb. fil.!”); an illustration (“Tab[ula] 94. DCCCXXV”) [53] (pl. 94) is also provided and it is part of the original material. Lacaita [46], incidentally indicated as “autotipo” the specimen by Reichenbach filius examined by Petrak ([24], p. 45) and preserved at W at that time (“H. N. W.”, according to the legend in ([24], p. 4)). In his works, Lacaita [46] often employed the term “autotipo”, which we can translate as “obvious lectotype”. Therefore, the designation by Lacaita [46] is valid and must be retained (Art. 7.11). However, note that our researches at W were useless, and it cannot be excluded that a new lectotype, possibily the illustration, should be designated in the future according to Art. 9.11.

*Distribution*—Species endemic to France (Maritime Alps) and northern and central Italy [6,19].

*Habitat*—Mountain pastures, rocky slopes, shrublands, pathways from 500 m to 1800 m a.s.l. [18].

**(8) *Cirsium scabrum*** (Poir.) Bonnet & Baratte, Expl. Sci. Tunisie, Cat. Pl.: 238. 1896 ≡ *Carduus scaber* Poir. (basion.), Voy. Barbarie 2: 231. 1789 ≡ Ca. *giganteus* Desf., Fl. Atlant. 2: 245. 1799, nom. illeg. (Art. 52.2) ≡ *Cirsium giganteum* Spreng., Syst. Veg. 3: 375. 1826, nom. ill. (Art. 58.1, Note 1) ≡ *Cnicus giganteus* Willd., Sp. Pl., ed. 4, 3(3): 1671. 1803, nom. illeg. (Art. 58.1, Note 1).—Lectotype (designated here): ”Numidia”, s.d., *Poiret s.n*. (P02837964 [digital image!], sub Ca. scaber).—https://science.mnhn.fr/institution/mnhn/collection/p/item/p02837964?listIndex=8&listCount=29.

**Figure 3:**
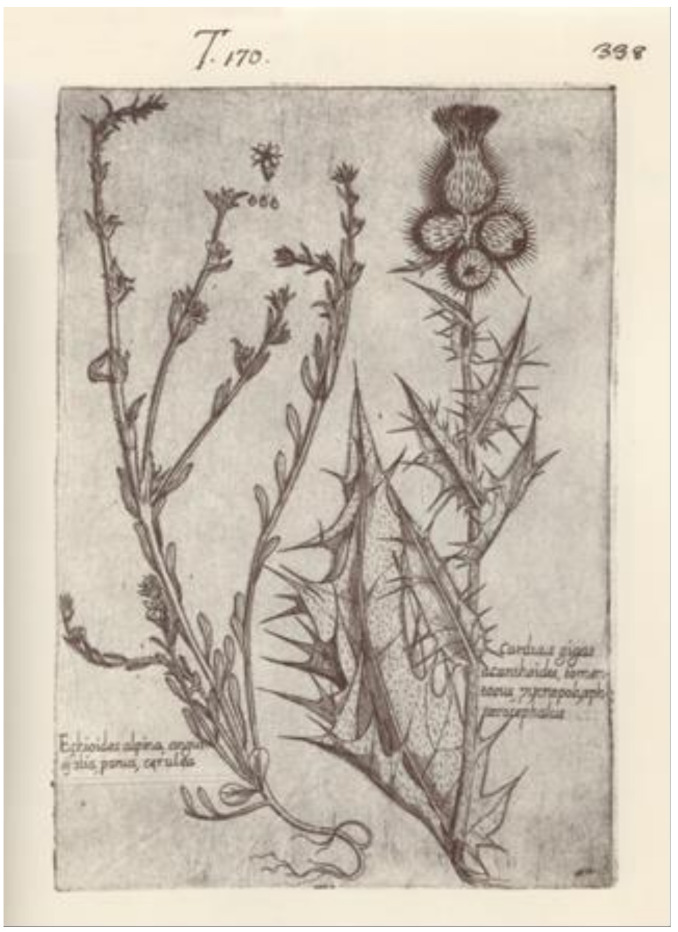
Lectotype of Ca. *gigas* Ucria (from the *Panphyton siculum*, plate 170, figure on the right side).

= *Carduus gigas* Ucria, Nuova Racc. Opusc. Aut. Sicil. 6: 255. 1793.—Lectotype (designated here): [illustration] “Carduus gigas acanthoides tomentosus, pycnopolysphaerocephalus” in Cupani [54] (Plate 170).—Figure 3.
= *Cirsium elatum* Tod., Index Seminum [Panormitani]: 25. 1858, nom. illeg. (Art. 53.1), non *Ci. elatum* Sauter, Flora 28: 130. 1845.—Lectotype (designated here): Italy, Sicily, Rifiesi [=Rifesi], fine di giugno [18] 52, *[A.] Todaro s.n.* (PAL, no. 10462!, sub Cnicus elatus).—http://147.163.105.223/zoomify/view_img.asp?ic=10462.
= *Cirsium giganteum* var. *macrocephalum* Lojac., Fl. Sicul. 2(1): 160. 1903.—Lectotype (designated by Aghababyan et al. [55] (p. 522)): Italy, Sicily,] Santa Cristina, s.d., *[A.] Todaro s.n.* (PAL, no. 10357!).—http://147.163.105.223/zoomify/view_img.asp?ic=10357).
= *Cirsium gigas* var. *eriophorum* Lojac., Fl. Sicul. 2(1): 160. 1903.—Lectotype (designated by Aghababyan et al. ([55], p. 522)): Italy, Sicily, s.d., *[A. Todaro] s.n.* (PAL, no. 10460!).—Image of the lectotype available at http://147.163.105.223/zoomify/view_img.asp?ic=10460.

*Notes on* Ca. scaber—Poiret [56] validly published this name by a Latin diagnosis (“Foliis amplexicaulibus lanceolatis dentato-spinosis supra scabris et viridibus; subtus tomentoso-albis, calyce inermi”; transl.: “[A Carduus] with leaves embracing, lanceolate, with spiny teeth, bristly and green above, lanate an whitish below, with unarmed involucres”), a description in French, and a taxonomic note. In particular, he hypothesized that the same plant could have been indicated by Tournefort by the polynomial “Cirsium orientale, cardui lanceolati folio flore purpurascente”. Finally, he indicated as habitat the stony and dry hills inhabited by the tribe of Nadis, probably corresponding to the area between North Tunisia and Algeria.

Only a single, relevant specimen is kept at P (F. Jabbour, *in litt.*). It originated from Poiret’s Herbarium and was later included in the collection of Moquin-Tandon. Poiret handwrote on the label “*Carduus scaber* (nobis)” and below (possibly later) “*Card. Giganteus* Desf. Atl.”. A different hand added, among other notes: “herb. Poiret ex Numidia” (a Latin term indicating the North-western Mediterranean Africa). Further pertinent material is lacking in the herbaria linked to Poiret: i.e., BR (F. Verloove, *in litt.*), FI (C. Nepi, *in litt.*), H (H. Väre, *in litt.*), UPS (M. Hjertson, *in litt.*) [57].

The name is the basionym for the accepted combination in *Cirsium*: our designation fully supports the current use of the name, on account of the large and not deeply divided leaves, heads in panicle more crowded upwards, the entire involucral bracts, which are appressed and tapering into an erect and short spine [5,8,18,26].

*Notes on* Ca. gigas—The protologue of *Ca*. *gigas* consisted of a short diagnosis (“CARDUUS Gigas foliis sinuato-spinosis, ramis floriferis brevibus”; transl.: “A Ca. very tall, with leaves sinuate-spiny, and heads brought by short branches) [58]. The author also reported a synonym from Francesco Cupani’s *Panphyton Siculum* (“Cup. Pamph.”) [54]. This work is actually “a collection of engravings of plants, animals and minerals”, remained uncomplete because of the premature death of the author [59]. However, much of his engravings (among which that of our interest) were published in 1713 (see Costa et al. [59]). The species was already reported by Cupani ([60], p. 37) with a slightly different polynomial (“Carduus gygas, pyramidalis, Acanthi foliis, tumentosis, pycnopolysphaerocephalus, flore albo”), noting also a purple-flowered variant. As the reference to *Panphyton siculum* by Ucria [58] is actually the citation of an illustration. This latter represents an element of the original material, probably the only one in existence. In fact, no specimen of Ucria’s was traced. The engraving of our interest represents the flowering stem of a thistle. Currently, Ca. *gigas* is regarded as a synonym of *Ci. scabrum*, and the above-said engraving (i.e., the proposed lectotype) is compatible with this identification: stem robust and rather unwinged, heads ovoid (only one at anthesis) in a racemiform array with short lateral branches, cauline leaves more or less plain, setose (or tomentose) on both the surfaces, lanceolate and acute, with spiny margins.

*Notes on* Ca. giganteus—Desfontaines [61] published the name Ca. *giganteus* by a diagnosis (“CARDUUS caule lanato; foliis cordatis, amplexicaulibus, sublobatis, superne hispidis subtus tomentosis, incanis, pedunculis uni ad trifloris”; transl.: “A *Carduus* with lanose stem; with leaves cordate, embrassing, sublobate, bristly above, covered by whitish tomentum below, with peduncles bearing up to three heads”), and a detailed Latin description. He also indicated the provenance (“Habitat in sepibus Algeriae”; transl.: “In the hedges of Algeria”) and provided an illustration [61] (plate 221), explicitly indicated in the protologue, which is part of the original material (“Tab[ula] 221”, https://www.biodiversitylibrary.org/item/7542#page/672/mode/1up). However, since Desfontaines [61] cited the validly published Ca. *scaber* as a synonym, Ca. *giganteus* is superfluous and illegitimate (Art. 52.2), and the type of this latter name is that of Ca. *scaber* (Art. 7.5). Analogously, the intended new combinations *Ci. giganteum* and *Cn. giganteus* are illegitimate as well (Art. 58.1).

*Notes on* Ci. elatum—Todaro [62] published this name in Latin with description, diagnosis, habitat and indication of the *loci classici*, all in southern Sicily: “*Monti di Rifesi, vicino Palazzo Adriano, fiume della Verdura sotto Ribera*”. He also pointed out that he had preserved the plant in the Herbarium Panormitanum under the name *Cnicus elatus*. We located a pertinent specimen at PAL (no. 10462, http://147.163.105.223/herbarium_vdetails_en2.asp?idmode=simple&id=22454). It includes a basal leaf and a cyme of mature heads, and was collected by Todaro himself at Ripesi, one of the *loci classici*, before the publication of the protologue. It is included in the fascicle of *Cn. elatus*, and, on the basis of the reference in the protologue, it could be even regarded as a syntype. Its examination fully supports the synonymization of *Ci. elatum* Tod. with *Ci. scabrum* (see Fiori [52]). Nevertheless, the name is illegitimate under Art. 53.1, because of the existence of the prior *Ci. elatum* by Sauter [63] (p. 130).

*Distribution*—A South-Western Mediterranean taxon, which indicates a species endemic to Western Mediterranean, occurring in Italy (Sardinia and Sicily included), France (Corse), Spain, Morocco, Algeria and Tunisia; locally adventitious in Germany [6], Portugal and even in North America [64].

*Habitat*—Wastelands, open woods, riparian vegetation, hedges, roadsides in the thermo-Mediterranean zone, preferably on sandy and acid soils, up to 1100 m a.s.l. [8,18,65].

**(9) *Cirsium spathulatum*** (Moretti) Gaud., Fl. Helv. 5: 202. 1829 ≡ *Cnicus spathulatus* Moretti (basion.), Giorn. Fis. Ser. 2, 5: 111. 1822 ≡ *Cirsium eriophorum* subsp. *spathulatum* (Moretti) Ces. in Cattaneo Not. Nat. Civ. Lombardia 1: 302. 1844 ≡ *Cirsium morettianum* Nym., Syll. Fl. Eur.: 24. 185. 1854–1855, nom. illeg. (Art. 52.2) ≡ *Ci. eriophorum* var. *spathulatum* (Moretti) Naeg., Syn. Fl. Germ. Helv. ed. 2 3: 989. 1845 ≡ *Cn. eriophorus* (L.) Roth subsp. *spathulatus* (Moretti) Arcang., Comp. Fl. Ital.: 404. 1882.—Neotype (designated here): Italy, “Comune nell’Italia settentrionale”, s.d., [ante 1819], *G. Moretti s.n*. (G-DC00486193 [digital image!], sub Cn. ciliatus W.).—https://www.ville-ge.ch/musinfo/bd/cjb/chg/adetail.php?id=335721&base=img&lang=fr).—“*Ci. insubricum* Moretti ex Bertol.”, Fl. Ital. [Bertoloni] 9(1): 25. 1853, nom. inval. (Art. 36.1b).

*Notes on* Ci. spathulatum—The name was published after March 1822 ([66], p. 46). Moretti [67] wrote the following diagnosis: “C[nicus] foliis profunde pinnatifidis, laciniis bipartitis, lineari-lanceolatis, apice spinosis, margine ciliatis, subtus tomentosis. Calycibus nudis, squamis spathulatis, apice spinosis” (transl.: “A *Cnicus* with leaves deeply pinnatifid, with linear-lanceolate segments, in turn almost divided in two part, spiny at the apex, ciliate along the margin, tomentose below. With glabrous heads, involucral bracts spathulate, with spiny points”). The protologue also includes a description, a reference to Villars et al. ([68], p. 45)—who misapplied the name Ca. *ciliatus* Murr. to the same plant described by Moretti–, the provenance (“in collibus ad meridiem Papiae”; transl.: “on the hills south of Pavia, northern Italy”), and a taxonomic note, all in Latin. Pertinent material is unfortunately lacking at BOLO, hosting specimens by Moretti (U. Mossetti, *in litt.*), or in other herbaria linked to Moretti [41]), i.e., C (O. Ryding, *in litt.*), FI (C. Nepi, *in litt.*), H (H. Väre, *in litt.*), PAD (R. Marcucci, *in litt.*), PAV (N.M.G. Ardenghi, *in litt.*) (no reply has been obtained by BASSA). A specimen at G (G00486193, http://www.ville-ge.ch/musinfo/bd/cjb/chg/adetail.php?id=335721&base=img&lang=fr) was sent by Moretti to Candolle in 1819 and was labelled by this latter author as “*Cnicus ciliatus* W.”. It is difficult to employ as lectotype, because it lacks any obvious link to the protologue or any clear association with the epithet “*spathulatus*” (even if the spathulate bracts are described in a separate label by Candolle). However, at present, it could be a suitable choice as neotype, because it is directly linked to Moretti, matches the protologue, and shows the typical features of the taxon (namely, the shape of the bracts).

*Notes on* Ci. morettianum—Nyman [69] proposed the name *Ci. morettianum* as a replacing name for the combination *Ci. spathulatus*, because, in his opinion, by this latter combination Gaudin [70] would indicate a different taxon as compared to that described by Moretti. However, as said above, on one hand *Ci. spathulatus* is homotypic with *Cn. spathulatum*; on the other hand, citing *Ci. spathulatum*, Nyman [69] published a superfluous and illegitimate name according to Art. 52.2, whose type is the type of *Cn. spathulatum* (Art. 7.5).

*Note on* Ci. insubricum—This name is reported by IPNI [27] as “*Cirsium insubricum* Moretti ex Bertol.”. However, Bertoloni [45] (p. 25) listed it as an unpublished name occurring in Moretti’s specimens, regarding it only as a synonym of *Cn. eriophorus*: it is invalidly published under Art. 36.1.

*Taxonomy*—The taxonomic value of this morph is controversial. Werner [5] recognized the specific rank, and Greuter [6] provisionally accepted it, while other authors (e.g., [18,19,71]) regard it as a subspecies of *Ci. eriophorum*. The morphological differences between *Ci. eriophorum* and *Ci. spathulatum* (especially regarding the presence of spiny appendages on the middle bract and the indumentum of the heads) are slightly and variable. In addition, no ecological or geographical segregation of the two taxa has been observed. Further studies might to include *Ci. spathulatum* in the specific variability of *Ci. eriophorum*; or, on the contrary, to show a closer affinity with other European taxa, such as *Ci. ligulare* Boiss. or *Ci. odontolepis* Boiss. ex DC.: therefore, we provisionally accept the specific rank.

*Distribution*—Taxon endemic and very local to Northern Italy and Switzerland [6,19].

*Habitat*—Pastures, grasslands, wood margins and disturbed environments on mountains [71].

**(10) *Cirsium tenoreanum*** Petr., Sched. Cirsiotheca Univ. 17: n. 168. 1921, nom. nov. pro *Ci. spurium* (DC.) Lacaita, Nuovo Giorn. Bot. Ital. n.s. 25: 119. 1918, comb. illeg. (Art. 53.1), non *Ci. spurium* (Del.) Del., Ann. Sci. Nat., Bot. sér. 2, 18: 149. 1842 ≡ *Ci. eriophorum* var. *spurium* DC. (basion.), Prodr. 6: 638. 1838.—Lectotype (designated by Lacaita [46] (p. 121), as “autotipo”): Italy, Abruzzes [=Abruzzo], Collines autour du Lac Fucin, 1832, *J. E. Duby s.n.* (G00486363 [digital image!]).—http://www.ville-ge.ch/musinfo/bd/cjb/chg/adetail.php?id=336417&base=img&lang=fr.

*Notes on* Ci. eriophorum *var.* spurium—Candolle [49] published the name of this new variety with a short diagnosis (“capitulis minoribus ovatis”; transl.: “with heads ovate and smaller [than in *Ci. eriophorum*]), relying on a specimen sent from Italy by Duby. The name was somewhat inspired by the Linnaean Ca. *eriophorus* var. *spurius* ([72], p. 824), indirectly cited by Candolle as a doubtful synonym by a reference to Linnaeus [73] (“An C. spurius Linn. hort. ups. 249?”), also cited by Linnaeus [72] himself in the protologue of Ca. *eriophorus* var. *spurius*. According to Del Guacchio and Iamonico [9], *Ci. eriophorum* var. *spurium* is to be regarded as the name of a new taxon, not having a basionym. On one hand, as those authors observed, the not validly published “*Ca. spurius*” (cited by Candolle) cannot be regarded as a basionym; on the other hand, however, it might be reasonable that the validly published Ca. *eriophorus* var. *spurius* is acceptable as basionym of the Candollean name under Art. 41.4. Actually, this article cannot be applied in any case, not even disregarding the taxonomic doubt by Candolle, because Ca. *eriophorus* var. *spurius* and *Ci. eriophorum* var. *spurium* definitely refer to different taxa, i.e., *Ci. ×gerhardtii* Schultz and *Ci. tenoreanum* respectively.

*Notes on* Ci. tenoreanum—The epithet is dedicated to the famous Italian botanist Michele Tenore (1780–1861). As explained by Lacaita [74], by this name Petrak [75] intended to replace *Ci. spurium* Lacaita, which had resulted a later homonym of *Ci. spurium* by Delastre [76] (p. 149) (cf. also Del Guachio and Iamonico [9]). The name by Petrak appeared for the first time in his series *Cirsiotecha Universa*, No. 168 in 1921; the same plant was also distributed later with number 198 (Scheuer [77]). According to Art. 30.8 (Ex. 12), the name was validly published in 1921. The printed label must be regarded as protologue, and the duplicates as obvious syntypes. The series is available in several herbaria: B, BM, C, G, K, LAU, PR, and W [57], and also M (http://indexs.botanischestaatssammlung.de/). We traced one duplicate of No. 168 in the personal herbarium of Lacaita himself, now kept at BM (BM001043049, http://data.nhm.ac.uk/dataset/collection-specimens/resource/05ff2255-c38a-40c9-b657-4ccb55ab2feb/record/1971813). The printed label reports the synonymy and the replaced name *Ci. spurium.* The specimen (and possibly every duplicate of No. 198) originated by the personal collections of Lacaita, gathered in 1914 by Donato Profeta in Abruzzo [46] (p. 121). It fully supports the current usage of the name, attributed to a species endemic to central and southern Italy, where it is common [18]. Finally, the nomenclatural type of the name is the type of his replaced name, i.e., *Ci. spurium*, and therefore of the basionym of this latter, i.e., *Ci. eriophorum* var. *spurium*.

*Distribution*—Species endemic to the Italian peninsula, where it is common [18,19].

*Habitat*—Pastures, grasslands, karst fields, paths, especially on limestones, from 1000 m to 1800 m a.s.l., rarely below [18] (pers. obs.).

**(11) *Cirsium vallis-demonii*** Lojac., Nat.. sicil. 3: 267. 1884 subsp. *vallis-demonii* ≡ *Ci. eriophorum* var. *vallis-demonii* (Lojac.) Fior, Fl. Italia [Fiori, Béguinot and Paoletti] 3: 367. 1904 (sub “Vallis-Daemonii”).—Lectotype (designated by Aghababyan et al. ([55], p. 522)): Italy, Sicily, Valdémone […] Mangalavite […], Julio 1882, *M. Lojacono s.n.* (G-BU).

=*Cirsium eriophorum* var. *involucratum* Coss., p. p. ([46], pp. 134–135).

—“Cirsium vallis-daemonis Lojac.”, var. orth.—“Cirsium vallis-demonis Lojac.”, var. orth.

**Figure 4:**
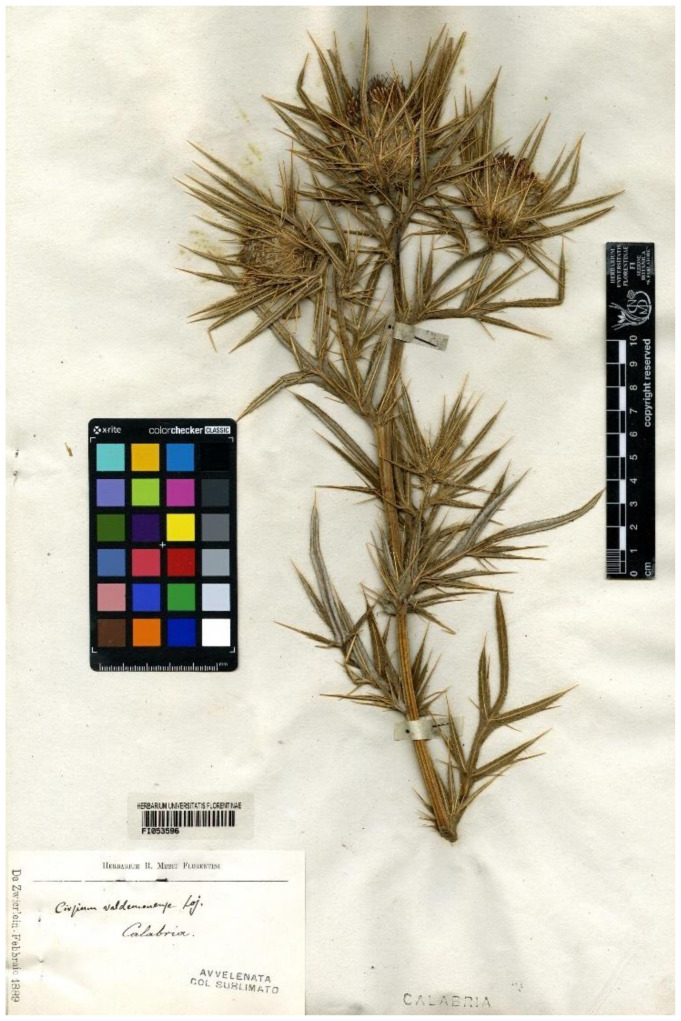
Lectotype of *Ci. eriophorum* var. *vallis-demonii* fo. *calabrum* Fiori (FI., barcode FI053596), by permission of the Curator.

**(12) *Cirsium vallis-demonii*** subsp. ***calabrum*** (Fiori) Del Guacchio, Bernardo, P.Caputo, Domina & Iamonico *comb. et stat. nov.* ≡ *Ci. eriophorum* var. *vallis-demonii* fo. *calabrum* Fiori, Fl. Italia [Fiori, Béguinot & Paoletti] 3: 367. 1904.—Lectotype (designated here)**:** Italy, Calabria, s.d., *F.V. Zwierlein s.n.* (FI, FI053596 (digital image!), sub *Cirsium valdemonense* Loj.)—Figure 4.

*Notes on* Ci. eriophorum *var.* vallis-demonii *fo.* calabrum—Fiori [50] described this form with a diagnostic phrase in Italian: “Fi. rosso-porporini od anche (*b. calabrum Nob.* = Cirs. Vall.-Daem. var. Lojac.) bianchi” (transl.: “flowers red-purplish [in the typical variety] or white ([var.] *b. calabrum* Nob[is] = *Ci. vallis-demonii* var. [unnamed variety] Lojac.”). Fiori [52] intended to validate at the form rank the taxon described by Lojacono Pojero [78]. This latter author, in fact, first observed that a specimen by F. V. Zwierlein from Calabria (Serra San Bruno) bore whitish or yellowish flowers. Nevertheless, his indications “Var. *floribus albis*” did not constitute a valid publication of a varietal name (Art. 23.6, Ex. 12). Therefore, according to Art. 9.4, we regard that specimen cited by Lojacono as original material for Fiori’s name, because of the direct reference to Lojacono. However, original material collected by Zwierlein in Serra San Bruno and directly examined by Fiori before the publication of the protologue is preserved at FI, together with further material explicitly revised by Fiori but collected after the protologue. In particular, FI053595 and FI053596 (this latter mounted on two sheets) were sent by Zwierlein to Florence in 1882. FI053595 is represented by a flowering branch (only one head is visible) and bears a label partly printed (“Da Zwierlein—Febbraio 1889”) and partly handwritten presumably by Zwierlein himself: “*Cirsium valdemonense* [sic!] Lojacono \ in tutta la Sila ed a Serra San Bruno”. FI053596 (first sheet) includes a flowering branch with three heads and a label identical to the other, but handwritten by Fiori: “*Cirsium valdemonense* Loj.\Calabria”. The second sheet bears a further flowering branch, without label. The printed date on the sheet and the adoption of the epithet “valdemonense”, not employed by Fiori later ([52,74,79] are convincing proofs that Fiori examined these specimens before the publication of the name. They show the typical features of *Ci. vallis-demonii* (e.g., the medium-sized heads, the numerous involucral leaves surrounding and exceeding them, the erect outer involucral bracts); the colour is obviously not well observable in dried material, but it was undoubtedly withish in vivo (see the inner flowers of the central head in FI052596\first sheet). We choose FI053596 as the lectotype of the Fiori’s name because it is more complete and bears the handwriting of the author.

*Taxonomy*—The Calabrian populations always show white-yellowish flowers [46] (L. Bernardo, pers. obs.); while in Sicily *C. vallis-demonii* has purplish flowers (G. Domina, pers. obs.). This character (i.e., purplish vs. whithish flowers) is taxonomical relevant, because—excluding obvious and sporadical albino individuals—it is constant within each species (cf. the dichotomous key in [3]). Besides, we found that at anthesis the middle involucral bracts are typically patent or divaricate in Sicilian populations, with purplish appendages; in the mainland populations only the inner bracts are divaricate, while the other ones are mostly erect or erect-patent, and the appendages are paler (E. Del Guacchio, pers. obs.). In addition, the leaves in var. *calabrum* would be typically less tomentose below [46], but this feature has been only partially verified by us on dried material (CAT, FI, PAL, PI). We note that, in the protologue, Fiori [52] reported the autonym form also for Calabria, but later he sharply kept the two ranges as distinct [79]. Also considering the complete separation of the ranges, we recognize the taxonomic value of fo. *calabrum*; nevertheless, following a more modern treatment, we prefer to propose here the subspecific rank for this taxon. The Strait of Messina and its adjacent mountains played an important role for subspecies differentiation: e.g., *Adenostyles alpina* (L.) Bluff & Fingerh. subsp. *nebrodensis* (Wagenitz and I.Müll.) Greuter (endemic to Sicily) vs. subsp. *macrocephala* (Huter, Porta & Rigo) Dillenb. and Kadereit (endemic to Calabria); *Anthemis cretica* L. subsp. *messanensis* (Brullo) Giardina & Raimondo (endemic to Sicily) vs. subsp. *calabrica* (Arcang.) R.Fern. (endemic to Calabria); *Aubrieta columnae* Guss. subsp. *sicula* (Strobl) M.A. Koch, D.A. German and R. Karl (endemic to Sicily) vs. subsp. *columnae* (endemic to Italy, from Lazio to Calabria); *Sesleria nitida* Ten. subsp. *sicula* Brullo and Giusso (endemic to Sicily) vs. subsp. *nitida* (endemic to Italy, including Calabria); *Thymus praecox* Opiz subsp. *parvulus* (Lojac.) Bartolucci, Peruzzi and Passal. (endemic to Sicily) vs. subsp. *polytrichus* (A.Kern. ex Borbás) Jalas (southern Europe, including Calabria) [1,19]. Future studies with new morphological and molecular observations might enlighten further differences.

*Distribution*—Endemic to Italy: the autonym subspecies grows in northern Sicily (Peloritani, Nebrodi and Madonie massifs), while the subsp. *calabrum* occurs in Calabria (Sila, Serra San Bruno) [18]. The presence more northward is not confirmed; a presumed specimen of this species collected on Pollino massif (CAT-003141!) is rather to be referred to *C. tenoreanum*.

*Habitat*—Mountain pastures, grassy lake shores, open woods, on limestones or granitic soils, from about 800 to 1500 m a.s.l. [18] (pers. obs.).

**(13) *Cirsium vulgare*** (Savi) Ten., Fl. Napol. 5: 209. 1835–1836 **≡** *Carduus vulgaris* Savi (basion.), Fl. Pis. 2: 241. 1798, nom. nov. pro Ca. *spinosissimus* Gerbi, Storia Nat. Nuovo Insetto 8: 9. 1794, non Ca. *spinosissimus* Walter, Walter, Fl. Carol.: 194. 1788, non Ca. *spinosissimus* Villars, Hist. Pl. Dauphiné 3(1): 11. 1788.—Lectotype (designated here): [illustration] “*Carduus spinosissimus*” in Gerbi [80] (Figure 1).—https://archive.org/stream/bub_gb_s7upD7SkUP0C#page/n11/mode/2up.

**Figure 5:**
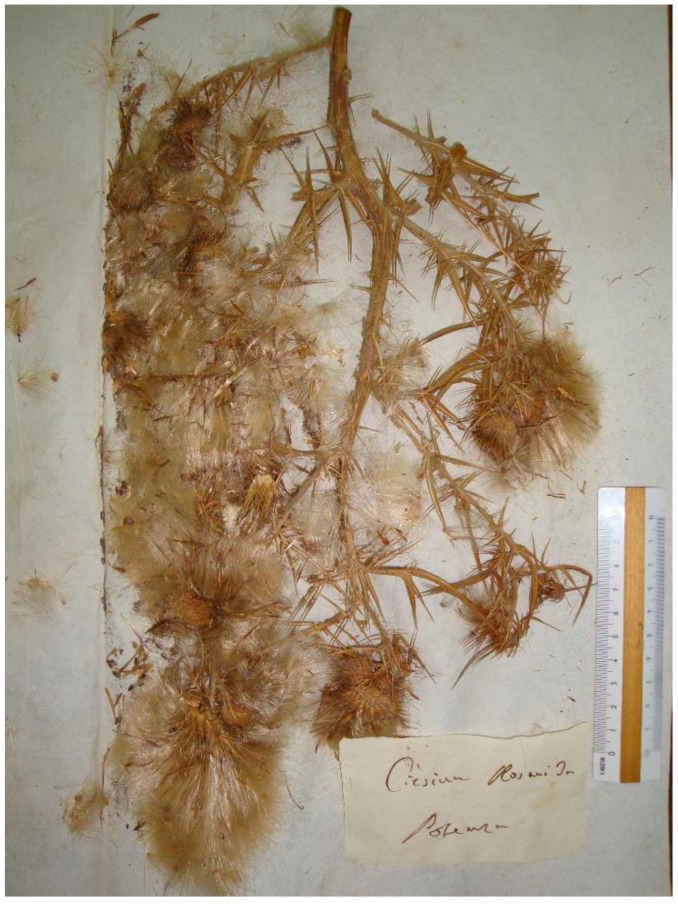
Neotype of *Cn. rosani* Ten. (NAP), by permission of the Director.

**Figure 6:**
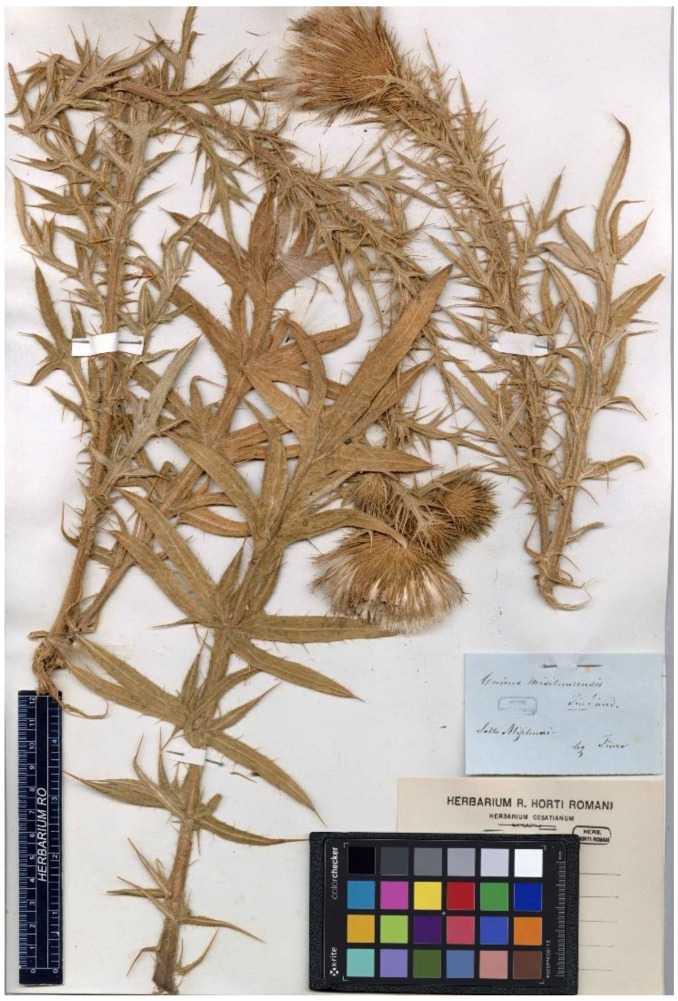
Lectotype of *Ci. misilmerense* Ces., Pass. & Gibelli (RO), by permission of the Curator.

= *Carduus lanceolatus* L., Sp. Pl. 2: 821. 1753 ≡ *Ascalea lanceolata* (L.) Hill., Herb. Brit. 1: 72. 1769 (cf. Art. 41.4) ≡ *Cirsium lanceolatum* (L.) Scop., Fl. Carniol., ed. 2, 2: 130. 1772, nom. illeg., non *Ci. lanceolatus* Hill., Herb. Brit. 1: 80. 1769 (Arts. 52.1–52.2) ≡ *Cnicus lanceolatus* (L.) Willd, Fl. Berol. Prodr.: 259. 1787 ≡ *Eriolepis lanceolata* (L.) Cassini in Cuvier Dict. Sci. Nat., ed. 2. [F. Cuvier] 41: 331. 1826.—Lectotype (designated by Talavera & Valdés [25] (p. 197)): Herb. Linnaeus, No. 966.1 (LINN!).—http://linnean-online.org/9800/.
= *Cirsium rosani* Ten., Index Sem. Hort. Bot. Neapol. 1830: 15. 1830 (sub “*Ci. rosani*”, cf. Art. 60.8 (a)) ≡ *Cnicus lanceolatum* subsp. *rosani* (Ten.) Arcang., Comp. Fl. Ital.: 403. 1882 ≡ *Ci. lanceolatum* subsp. *rosani* (Ten.) Arcang., Comp. Fl. Ital., ed. 2: 723. 1894.—Neotype (designated by Lacaita [46] (p. 125)): Italy, Basilicata, Potenza, s.d., *F. Rosano? s.n.* (NAP, collection Tenore!).—Figure 5.
= *Cirsium crinitum* Boiss. ex DC., Prodr. 7(1): 305. 1838 ≡ *Ci. lanceolatum* subsp. *crinitum* (Boiss. ex DC.) Bonnier & Layens, Tabl. Syn. Pl. Vasc. France: 175. 1894 (cf. p. VIII of the same work) ≡ *Ci. vulgare* subsp. *crinitum* (Boiss. ex DC.) Arènes, Bull. Soc. Franç. Echange Pl. Vasc. 1: 21. 1948.—Lectotype (designated by Talavera & Valdés [25] (p. 201)): France, Narbonne, 1828, *E. Requien s.n.* (G, G-DC00493688 [digital image!], sub Ci. echinatum).—http://www.ville-ge.ch/musinfo/bd/cjb/chg/adetail.php?id=407321&base=img&lang=fr.
= *Cirsium misilmerense* Ces, Pass. & Gibelli, Comp. Fl. Ital. 2(21): 483. 1878.—Lectotype (designated here): Italy, Sicilia, Sotto Misilmeri, s.d. *s.n.* (RO!).—For an image of the lectotype, see Figure 6.
= *Cirsium cardoleonis* Lojac., Fl. Sicul. 2(1): 158. 1903—Lectotype (designated by Aghababyan et al. [52] (p. 521)): Italy, Sicily, Santa Cristina, July 1873, *M. Lojacono-Pojero s.n*. (PAL, no. 10188!).—http://147.163.105.223/herbarium_vdetails_en2.asp?idmode=simple&id=22320.
= *Cirsium dubium* Lojac., Fl. Sicul. 2(1): 155. 1903.—Lectotype (designated by Aghababyan et al. [55] (p. 521)): Italy, Sicily, Regalbuto, s.d., *Todaro s.n.* (PAL, no. 10475!, sub Cn. lanceolatus var. incanescens).—http://147.163.105.223/herbarium_vdetails_en2.asp?idmode=simple&id=22476.
= *Cirsium lanceolatum* var. *subbipinnatum* Lojac., Fl. Sicul. 2(1): 155. 1903.—Lectotype (designated by Aghababyan et al. [55] (p. 522)): Italy, Sicily, Is. Eolie Alicuri, s.d., *M. Lojacono* (PAL, no. 10370!).—http://147.163.105.223/herbarium_vdetails_en2.asp?idmode=simple&id=22387.
= *Cirsium lanceolatum* var. *tenuispinum* Lojac., Fl. Sicul. 2(1): 155. 1903 (sub “*tenuispinus*”).—Lectotype (designated by Aghababyan et al. [55] (p. 522)): Italy, Sicily, S Martino, s.d., *M. Lojacono-Pojero s.n.* (PAL, no. 10367!).—http://147.163.105.223/herbarium_vdetails_en2.asp?idmode=simple&id=22379.
= *Cirsium vulgare* var. *longespinosum* Rouy, Fl. France [Rouy & Foucaud] 9: 21. 1905, nom. illeg. (Art. 52.1).—Lectotype (designated here): Italy, Sicily, Palermo sotto la Grazia, Aug [s.d., s.a.], *A. Todaro n. 528* (PAL, no. 10364!, sub Ci. lanceolatum All. var. firmus).—http://147.163.105.223/herbarium_vdetails_en2.asp?idmode=simple&id=22375.
= *Ci. lucanicum* Lojac., Nat. sicil. 3: 283. 1884—Type:—Not designated (see Domina et al. [81]).

“*Ci. lanceolatum* var. *vulgare* Naeg.”, Syn. Fl. Germ. Helv., ed. 2, 3: 990. 1845, nom. inval.

“*Ci. vulgare* (Savi) Airy-Shaw”, Repert. Spec. Nov. Regni Veg. 43: 304. 1938, isonym (Art. 6, Note 2).

“*Ci. vulgare* (Savi) Petr.”, Sched. Cirsiotheca Univ. 4: n. 33. 1912, nom. prov. (Art. 36.1). By this provisional name, Petrak indicated the taxon correctly named *Ci. italicum* [82].

*Notes on* Ca. spinosissimus—In a rare booklet, Gerbi ([80], p. 9–10) validly published the name Ca. *spinosissimus*. IPNI [27] reports on p. 9 as that of the protologue, but the plant was named and described in Italian already at p. 8, also providing a detailed description, and an illustration, i.e., “*Figure I*” by Gerbi [80], that is original material for the name. Unfortunately, the Gerbi name is a later homomyn of Ca. *spinosissimus* Walter, and so illegitimate under Art. 53.1. Since no specimens constituting original material were traced, we would designate the Gerbi’s image as the lectotype of the name *Ca. spinosissimus*. The illustration depicts the plant nowadays called *Ci. vulgare* (Savi) Ten.

*Notes on* Ca. vulgaris—The name was published by Savi ([83], p. 241) after 22 January (D’Antroccoli and Peruzzi [84]) by a diagnosis (“*Carduus foliis semi-decurrentibus, bifariam pinnatifidis, calycibus solitariis ovatis, sublanatis*”; transl.: “A *Carduus* with leaves half-decurrent, bi-pinnatifidous, with heads solitary, ovate, almost lanose”) taken directly from the protologue of Ca. *spinosissimus* by Gerbi ([80], pp. 9–10). Moreover, Savi [83] explicitly wrote that he intended Ca. *vulgaris* as an avowed substitute (nomen novum) for the later homonym Ca. *spinosissimus* Gerbi. As a consequence, both Savi’s name (Art. 7.4) and obviously its combination in *Cirsium* [85] (p. 209) are homotypic with it.

*Notes on* Ci. rosani—Contextually with the name *Ci. lobelii*, Tenore [48] (p. 14) published also a new species in *Cirsium*, dedicating it to his correspondent Francesco Antonio Rosano (1779–1843), who first gathered the plant in Basilicata (a region of southern Italy). The protologue includes a Latin description and the provenance (“In arvis Lucaniae prope *Potentiam*”, i.e., “in the fields of Basilicata near the city of Potenza”). Also in this case, Lacaita [46] (p. 125) indicated an “autotipo” from Tenore’s herbarium, a single specimen gathered in Potenza (Basilicata) by Rosano (Figure 5). Probably, it is original material; however, there is no definitive proof on the matter. Lacking certain original material, the designation by Lacaita must be retained. After the examination of the available material and the original description, and according to our broad circumscription of *Ci. vulgare*, *Ci. rosani* can be considered a heterotypic synonym. The plate in *Flora napolitana* [48], published slightly later than the protologue, supports this identification.

*Notes on* Ci. misilmerense—This name was published by Cesati et al. [86], who reported the diagnostic features of the taxon within a dichotomic key (“Brattee assai più brevi del capolino, squame dell’invoglio appressate e terminanti in uno spino diritto e pungente”; transl.: “Bracts much shorter than the capitulum, phyllaries appressed, each one with a terminal straight and stinging spine”), the indication of the *locus classicus* (“*Sotto Misilmeri (Sicilia)*”), and of the unpublished name “Cnicus misilmerensis *Tineo! ined.*”. The exclamation mark infers that the new species was described on the basis of a specimen of Tineo’s seen by Cesati. We found this specimen (original material) in the Herbarium Cesatianum at RO. It bears a well-preserved plant and the original label by Tineo “*Cnicus misilmerensis Tin.! ined.*|*Sotto Misilmeri*|*leg. Tineo*”. Another interesting specimen by Tineo is at PAL (no. 84936), but possibly it was not examined by the authors of the name. Even if regarded, especially in the past, as a distinct [78,87,88] or a critical species [18,89], *Ci. misilmerense* is nowadays mostly included in the variability of *Ci. vulgare* subsp. *crinitum* [6,19].

*Notes on* Ci. lucanicum—Lojacono Pojero [78] described this species on the basis of an apparently single exsiccatum collected by Gasparrini from Lucania (currently Basilicata region in southern Italy). In fact, as in the protologue, Lojacono Pojero [78] wrote “in specimine meo” (“in my specimen”), this could be interpreted as the only element on which he based the description; however, it is not considerable as the holotype [90]. Lojacono Pojero [78] did not ascribe the name to Gasparrini, but to himself: “Cirsium lucanicum MIHI. GASPARRINI ined. in Pl. sicc. ex Lucania”. Unfortunately, we did not find any pertinent specimen at FI, NAP, MS, MPU, PAV, and PAL. The protologue includes a Latin description and an Italian diagnosis to distinguish *Ci. lucanicum* from *Ci. italicum* and *Ci. lobelii* sensu Lojacono (=*C. tenoreanum*). According to modern views [6,18], *Ci. lucanicum* is a synonym of *Ci. vulgare* subsp. *crinitum*, which we include in *Ci. vulgare*. This statement is possibily based also on the taxonomic doubt expressed by Lojacono, who hypothesises that *Ci. lucanicum* could be *Ci. rosani.* Nevertheless, according to the protologue, *Ci. lucanicum* has not decurrent leaves, and this detail would definitely exclude the synonym. In absence of original material and considering this doubt about the synonymization, we refrain to typify this name at present.

*Notes on* Ci. vulgare *var.* longespinosum—Rouy [91] published this name citing Todaro’s *Flora Sicula Exsiccata* n. 528, a synonym by Lamotte, a diagnosis in French (allowed at the time according to Art. 39.1), and some localities (at p. 22). Apparently, Rouy himself attributed the name to Todaro. However, as far as we known, this latter author never employed that epithet, not even in his exsiccata. M. Thiébaut (LY) (*in litt.*) informed us that Rouy [91] only cited Todaro’s specimen but didn’t hold it in his own herbarium. As Rouy [91] cited the previous and legitimate name *Ci. lanceolatum* var. *horridulum* Lam., whose epithet ought to have been adopted at varietal rank, its name is definitely superfluous and then illegitimate under Art. 52.1. Nevertheless, citing *Flora Sicula Exsiccata* n. 528, Rouy [91] actually indicated a type different from that of Lamotte, and we can treat it as the name of a new taxon (Art. 7.5, case b). On two specimens from Rouy’s herbarium, originating from Basses Pyrénées and Aude—localitites reported in the protologue ([91], p. 22) –the author himself wrote by hand “*longespinosum*”: LY0718280 and LY0718281. These specimens were collected before the protologue and are undoubtedly original material for the name. However, as a further consequence of citing *Flora Sicula Exsiccata* n. 528, the specimens of this series number are syntypes, which are preferred material for lectotypification (Art. 9.12), also those not seen by the author himself (Art. 9.4). Therefore, we would propose a pertinent specimen at PAL; other syntypes would be preserved in the herbarium cited by Stafleu and Cowan [92] (e.g., K000778051). The proposed lectotype was collected by Todaro near Palermo and is labelled as *Ci. lanceolatum* All. var. *firmum*” (see below for *Cn. firmus*); it includes a flowering stem of *Ci. vulgare* (stem completely winged, heads larger than 20 mm).

*Notes on* Cnicus firmus—It is currently believed that the taxon named *Cn. firmus* C.Presl ([93], p. 107) is to be included in *Ci. vulgare* [6,18]. However, the examination of original material (PRC!) suggested that the taxon must be referred to another genus, and therefore that name is not treated here.

*Taxonomy*—This is a highly variable species. The infraspecific taxa recognized in modern times by several authors (e.g., [18,19]), i.e., *C. vulgare* subsp. *crinitum* (DC.) Arènes and *C. vulgare* subsp. *silvaticum* (Tausch) Arènes, are only preliminarily accepted by Greuter [6], Shin and Greuter [94] and are rejected by most scholars worldwide (e.g., [1,5,8,42,84,95,96]). According to the living or dried material examined by us and in the absence of any convincing discontinuity among the three presumed subspecies in terms of morphology, ecology or phytogeography, we agree with these authors.

*Distribution*—Widespread and common from the Mediterranean Basin and Europe to Asia, but naturalized worldwide [97,98].

*Habitat*—Clearings, riparian vegetation, hedges, fields, very often synanthropic (roadsides, ruderal environments, pathways, etc.), on rich and nitrified soils [97].

## 4. Conclusions

Nomenclatural studies play a central role in systematics and they should be regarded as essential and preliminary for any taxonomic assessment. On one hand, our contribution on *Cirsium* sect. *Eriolepis* in Italy allowed us to re-evaluate one neglected taxon (i.e., *Cirsium vallis-demoniii* f. *calabrum*) and to ascertain most synonymies for the correct interpretation of the names. On the other hand, we showed that, in some cases, previous synonymizations were erroneous (e.g., *Cnicus firmus*) or very doubtful. In addition, the examination of the original material of names linked to critical taxa (e.g., the three presumed subspecies of *Cirsium vulgare*) suggests that further research should be carried out before accepting taxonomic conclusions. Finally, some overlooked lectotypifications (e.g., *C. morisianum*) were brought to light.
